# Fall prevention by reactive balance training on a perturbation treadmill: is it feasible for prefrail and frail geriatric patients? A pilot study

**DOI:** 10.1007/s41999-023-00807-9

**Published:** 2023-06-13

**Authors:** Ulrike Sonja Trampisch, Alexander Petrovic, Diana Daubert, Rainer Wirth

**Affiliations:** Department of Geriatric Medicine, Marien Hospital Herne, Ruhr University Bochum, Hölkeskampring 40, 44625 Herne, Germany

**Keywords:** Feasibility studies, Cognition, Frail elderly, Aged, Aged, 80 and over, Exercise, Accidental falls

## Abstract

**Purpose:**

Prefrail and frail geriatric patients are at high risk of falling. Perturbation-based balance training on a treadmill appears to be highly effective, but there are no studies in prefrail and frail geriatric hospital patients. The aim of the work is to characterize the study population in whom reactive balance training on a perturbation treadmill was feasible.

**Methods:**

The study is recruiting patients with at least one fall event in the past year (age ≥ 70). The patients complete a minimum of 60-min treadmill training with/without perturbations on at least 4 occasions.

**Results:**

Until now, 80 patients (mean age 80 ± 5) took part in the study. More than half of the participants had some cognitive impairment with < 24 pts. (median MoCA 21 pts.), 35% were prefrail and 61% were frail. The drop-out rate was initially 31% and was reduced to 12% after adding a short pre-test on the treadmill.

**Conclusion:**

Reactive balance training on a perturbation treadmill is feasible for prefrail and frail geriatric patients. Its effectiveness in fall prevention in this population needs to be proven.

**Trial registration:**

German Clinical trial register (DRKS-ID: DRKS00024637 on 24.02.2021).

## Key summary points

**Aim:**

The aim of the work is to characterize the study population in whom reactive balance training on a perturbation treadmill was feasible.

**Findings:**

More than half of the participants had some cognitive impairment with < 24 pts. (median MoCA 21 pts.), 35% were prefrail and 61% were frail. The drop-out rate was initially 31% and was reduced to 12% after adding a short pre-test on the treadmill.

**Message:**

Reactive balance training on a perturbation treadmill is feasible for prefrail and frail geriatric patients.

## Background

Depending on their degree of frailty, geriatric patients are at risk of falling [1]. While various training concepts demonstrated a moderate reduction of future falls [2], reactive balance training with unannounced perturbations appears to be highly effective in preventing falls. The novel training approach of perturbation training demonstrated approximately a 50% reduction in falls over a 12-month follow-up period after one session with 24 perturbations in relatively healthy older individuals using a gait parcour [3]. Previous studies have examined the concept of perturbation-based balance training in community-dwelling elderly subjects in good functional condition [3–9]. Only a few studies have been performed using a perturbation treadmill. A meta-analysis of different types of perturbation training showed a significantly 46% lower incidence of falls in the intervention group compared to the control group [4]. Another meta-analysis presented perturbation training as an optimal intervention to improve reactive balance in older adults [10]. Therefore, perturbation based balance training appears to be more effective than other fall prevention programs [11]. These studies all show clear evidence for healthy older adults. So far, there have been no such fall prevention studies on fall-prone geriatric hospital patients with comorbidity, prefrailty, frailty, and deficits in balance, gait, strength, and cognition, even though they are at highest risk of falls and fall related injuries. Thus, it is not yet clear, if this training concept is feasible and effective in this cohort at all. Fear of falling, for example, may play an important role while walking on an unstable ground and may lead to discontinuation and rejection of participation [12]. In addition, it is questionable, if subjects with cognitive impairment understand the training instructions and are able to follow the training protocol. In presenting the available data, it is therefore not a primary question of what training intensity is possible, but rather whether training on the treadmill with perturbations is even possible for geriatric patients and up to which degree of physical and cognitive frailty. The aim of the present work is to characterize the geriatric study population in whom reactive balance training on a perturbation treadmill was feasible in this pilot study.

## Methods

### Study design and participants

This feasibility study is an interim analysis of a randomized controlled trial (RCT) that started in October 2019. Due to the pandemic, the recruitment is still ongoing and the study will be finished by the end of the year 2023. The assessor blinded RCT is undertaken in the acute care geriatric hospital department of Marien Hospital Herne, Ruhr University Bochum, Germany. Participants are admitted to the geriatric acute care ward as inpatient or day clinic inpatient. The RCT is recruiting patients with at least one fall event in the past year. Further inclusion criteria for the analysis were 70 years or older, patient is able to walk, patients’ weight < 150 kg and height < 185 cm, ability to participate in the study, and the patient is physically able to complete the training on the treadmill. Exclusion criteria were total hip replacement less than 4 weeks ago, severe pain when walking, severe paralysis, leg amputees, and palliative situation. We assigned patients randomly (1:1 using a randomization software) to the intervention or control group. Patients completed a training of at least a total of 60-min on minimum 4 occasions on the treadmill (15 min per session) within 1–2 weeks, either with treadmill-based perturbation training (intervention group) or conventional treadmill training without perturbations on the identical device (control group). The training was conducted and closely monitored by physiotherapists. Therapist adjust walking speed and duration of the training (control group) and frequency, directions and strength of perturbations (intervention group) according to the patients’ individual capability, acceptance and training progress. In the intervention group, each training session consisted of an individual amount of random right/left/forward/backward unannounced platform translations while walking. In order to adequately address the patient's initial fear, the therapist stood sideways on the treadmill to offer the patient a supporting hand in case of uncertainty. As the training progressed and the anxiety diminished, the therapist stopped standing on the treadmill. We use cross sectional data from baseline. We also asked the therapists for feedback regarding the training with the perturbation treadmill. These were non-structured interviews without a systematic approach with validated tools. We define feasibility as a completed intervention of at least 60 min of treadmill training regardless of training intensity.

The ethical committee of Ruhr University Bochum approved the study protocol (no 19-6616-BR approved on 07.06.2019). The study is registered at German Clinical trial register (DRKS-ID: DRKS00024637 on 24.02.2021).

### Patients’ characteristics

Geriatric assessment was routinely performed at hospital admission including patients’ date of birth and sex, height and weight. Participant’s age was calculated for the day of hospital admission. Activities of daily living were determined using the Barthel-Index (BI) [13]. Hand grip strength was assessed with a hand dynamometer, and is defined as the maximum value from three attempts with the dominant hand (if not possible, non-dominant hand). Cognitive function was assessed with the German Montreal Cognitive Assessment (MoCA) [14]. If the assessment with MoCA could not be fully recorded for non-cognitive reasons, i.e. in case of severe visual impairment, the result was extrapolated according to the amount of questions answered. The total MoCA score uncorrected for education was used for analyses. The Short Physical Performance Battery (SPPB) assesses lower extremity physical performance status to evaluate functional capability [15]. Fear of falling was measured with the Falls Efficacy Scale (FES-I) [16, 17]. Depressive symptoms were diagnosed using Depression in Old Age Scale (DIA-S) [18]. FRAIL scale was used to identify persons at risk of frailty [19]. Risk of malnutrition was measured with the Mini Nutritional Assessment Short-Form (MNA-SF) [20]. SARC-F questionnaire was used to identify persons at risk of sarcopenia [21]. For assessment of global mobility and future follow-up, we used the Parker Mobility Score [22].The treatment report for the treadmill training includes total records, total days, total treatment duration in hh:mm, walking speed (km/h), and amount of perturbations (only intervention group).

### Treadmill

**Fig. 1:**
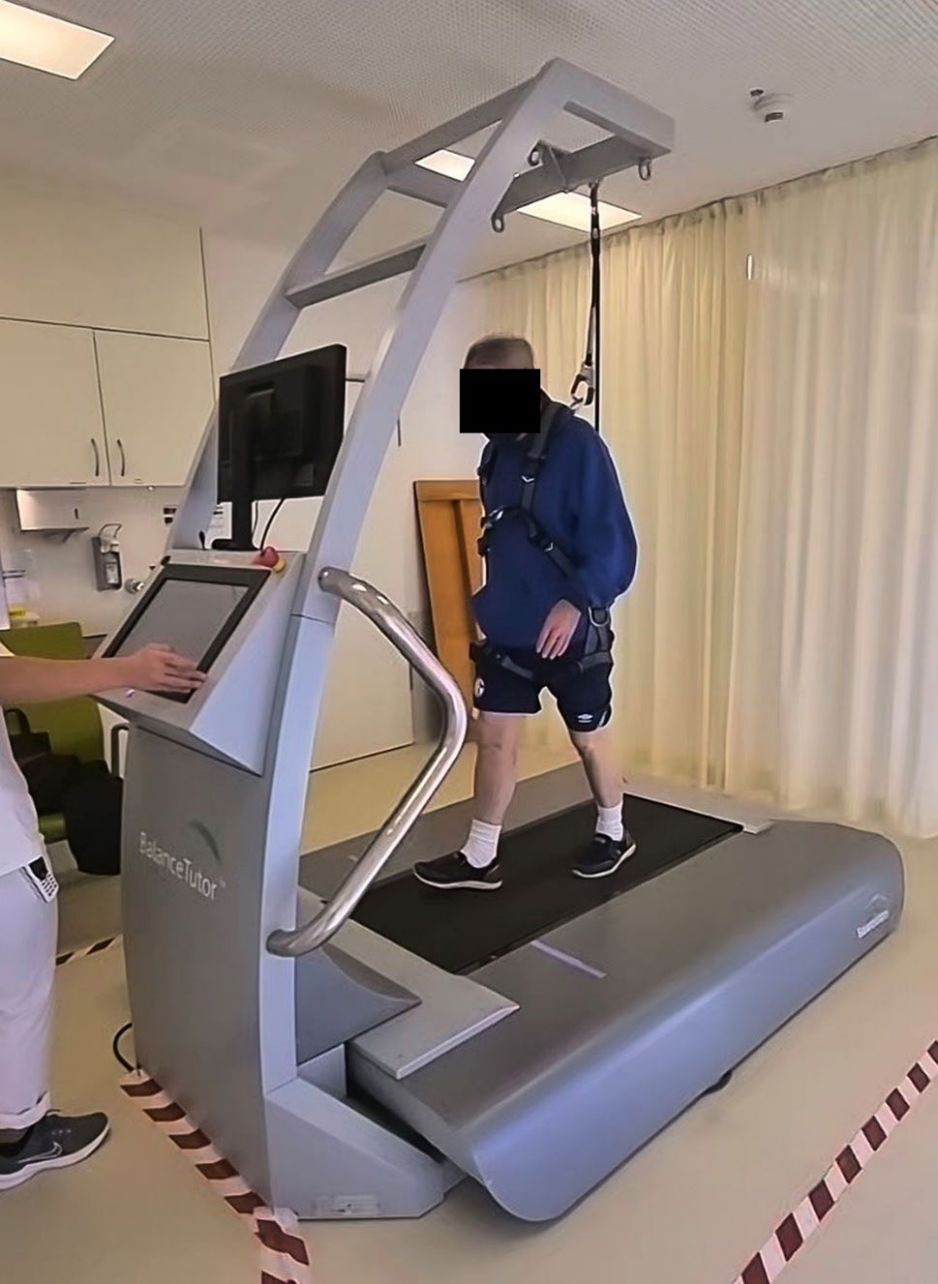
Patient walking on the perturbation treadmill secured by a safety harness

The treadmill (Balance Tutor™ BT100, MediTouch, Israel) is a rehabilitation device for the training of reactive balance. Perturbation movements on the treadmill are possible in all four horizontal directions. Participants can experience and train unexpected stumbling and slipping without the risk of falling, because a safety harness secures them (Fig. 1).

### Statistical analysis

The statistical analysis was completed using SPSS statistical software (IBM SPSS, Version 28.0). Means and standard deviations (SDs) were used for continuous data. Categorical variables are demonstrated as n (%).

## Results

### Characterization of study population

The study population comprised 80 older participants aged between 70 and 90 years (mean age 80 ± 5 years) with 56 (70%) women. 49 (61%) participants are classified as “frail” (Frail-Scale). More than half of the patients demonstrate cognitive impairment (mean MoCA 21 ± 4 pts.). The average SPPB is 9 points (± 3). Further characteristics of study population are shown in Table 1.

**Table 1:** Characteristics of the study participants (n = 80)

	n (%) or mean (sd)	Min	Max
Age in years	80 ± 5	70	90
Female	56 (70%)		
Height (m)*	1.63 ± 0,1	1.42	1.82
Weight (kg)*	72 ± 15	40	125
Body Mass Index (kg/m^2^)*	27 ± 5	17	45
Intervention group (n)	46 (56%)		
Geriatric assessment			
Barthel-Index (median) (pts.)	70	45	100
Hand grip strength max value (kg)*	20 ± 9	4	40
Montreal Cognitive Assessment (pts.)*	21 ± 4	8	29
Parker Mobility Score*(pts.)	7 ± 2	2	9
Short physical performance battery (pts.)*	9 ± 3	1	12
Fear of falling (short FES-I) (pts.)	12 ± 5	7	25
Depression in old age Scale (DIA-S) (pts.)			
No depressive symptoms	42 (51%)		
Mild to moderate depressive symptoms	11 (14%)		
Severe depressive symptoms	28 (35%)		
Frail scale			
Robust	3 (4%)		
Pre-frail	28 (35%)		
Frail	49 (61%)		
Mini Nutritional Assessment (MNA-SF)*			
Normal nutritional status	19 (24%)		
At risk of malnutrition	46 (58%)		
Malnourished	14 (18%)		
Sarcopenia (SARC-F)			
Low risk	30 (37%)		
High risk	50 (63%)		
Treatment report (treadmill)			
Total records	14 ± 5	5	30
Total days	7 ± 2	3	12
Total treatment duration in hh:mm	1:46 ± 0:39	1:00	3:46
Maximal walking speed (km/h)	1.3 ± 0.5	0.2	5.4
Total perturbations^+^	720 ± 276	352	1508

*1–2 missing values; ^+^only intervention group (n = 44)

### Drop-outs

After having recruited 38 patients for the trial, we realized a relatively high drop-out rate of 31% (n = 12). In particular, patients turned out not to be eligible for the study during first training session, because they were too frail to walk several minutes on the perturbation treadmill. To determine definitive eligibility for participation in the study before randomization, we added a short pre-test (1–3 min.) on the treadmill without perturbations. Doing so, we reduced the drop-out rate for the remaining 62 patients to 12% (n = 8). In total, we had 25% drop-outs (n = 20) with 45% (n = 9) in the control and 55% (n = 11) in the intervention group (*p* = 0.6). Reasons for drop outs ranged from not suitable, patients fell short of the minimum training intervention duration of 60 min total, or unclear reasons (Table 2). Patients were found to be unsuitable if, for example, the training was only possible with permanent restraint of the patient by a therapist, the patient had dyspnea, or pain occurred due to a vertebral body fracture. One patient from the intervention refused training on the treadmill altogether.

**Table 2:** Reasons for patients’ drop out (n = 20)

Reason for drop out (n)	Intervention group	Control group
Not eligible	5	5
Treatment duration (60 min) not met	2	4
Unclear reason	4	–
All	11	9

### Treatment report

Patients trained on average during 14 (± 5) session on 7 (± 2) days, which means that training was mostly performed with a short rest in between single training units of 7–8 min. The average training time was 01:46 hh:mm (± 0:39). The total perturbation count was 720 (± 276) per person (average 6.8 perturbation per minute).

### Feedback from therapists

In a personal unstructured conversation with the author UST the therapists value the training on the perturbation treadmill as an effective and safe training for frail patients. They also reported that some patients were afraid of falling, even though they were secured by the safety harness. At first glance, the perturbation treadmill is an impressive training equipment (see Fig. 1), and the therapists need to be sensitive to anxious patients in order to motivate them to try walking on the perturbation treadmill. In the case of cognitively impaired patients, the implementation of the therapists’ instructions is sometimes challenging.

## Discussion

In our acute care geriatric hospital, we use the perturbation treadmill since February 2019. We conceptualized a pilot study for the area of application with frail geriatric patients who are inpatient or day clinic inpatient in acute treatment. Our patients’ functionality varies broadly from bedridden, to only a few steps possible with a walker to independently mobile. Patients with the highest risk of falling, who might probably benefit from perturbation-based balance training on a treadmill, are mostly in a poor functional condition. Until now, no study on reactive balance training on a perturbation treadmill exist in prefrail and frail geriatric patients with comorbidities and cognitive impairment. Patients with cognitive impairment required more intensive and possibly repeated instruction for the task, but were then able to cope with the training. In this cohort of 80 patients we found that patients with cognitive impairment, prefrailty and frailty are able to participate in fall prevention trials using a perturbation treadmill. A high degree of training individualization appears to be necessary for these patients, which needs to be considered for upcoming studies. The number and reasons for dropping out are quite balanced between the intervention group and control group. Overall, there were slightly more drop outs in the intervention group, which can be considered a coincidence given the low number of drop outs overall. The higher number of unknown reasons in the intervention group is somewhat noticeable and could be due to the perturbations. However, this was not explicitly asked and can only be assumed speculatively. We conclude that reactive balance training on a perturbation treadmill is feasible for fall-prone frail geriatric hospital patients.

### Limitations

This study has limitations beside its single center design. We did no systematic eligibility screening of all our hospital patients and do not have sufficient data from drop out patients, so a non-responder analysis is not possible. Moreover, the feedback from therapists were reported as non-structured interviews without a systematic approach with validated tools and may be biased.

## Conclusion

Perturbation-based balance training on the treadmill is feasible for frail geriatric patients. Its effectiveness in fall prevention needs to be proven.

## Data Availability

The datasets used and/or analysed during the current study are available from the corresponding author on reasonable request.
